# Creating Anti‐Chiral Exceptional Points in Non‐Hermitian Metasurfaces for Efficient Terahertz Switching

**DOI:** 10.1002/advs.202402615

**Published:** 2024-05-17

**Authors:** Zhongyi Yu, Weibao He, Siyang Hu, Ziheng Ren, Shun Wan, Xiang'ai Cheng, Yuze Hu, Tian Jiang

**Affiliations:** ^1^ College of Advanced Interdisciplinary Studies National University of Defense Technology Changsha 410073 P. R. China; ^2^ Institute for Quantum Science and Technology College of Science National University of Defense Technology Changsha 410073 P. R. China

**Keywords:** active non‐Hermitian metasurface, loss‐induced anti‐chiral exceptional points, terahertz switching, ultrafast photonics

## Abstract

Non‐Hermitian degeneracies, also known as exceptional points (EPs), have presented remarkable singular characteristics such as the degeneracy of eigenvalues and eigenstates and enable limitless opportunities for achieving fascinating phenomena in EP photonic systems. Here, the general theoretical framework and experimental verification of a non‐Hermitian metasurface that holds a pair of anti‐chiral EPs are proposed as a novel approach for efficient terahertz (THz) switching. First, based on the Pancharatnam–Berry (PB) phase and unitary transformation, it is discovered that the coupling variation of ±1 spin eigenstates will lead to asymmetric modulation in two orthogonal linear polarizations (LP). Through loss‐induced merging of a pair of anti‐chiral EPs, the decoupling of ±1 spin eigenstates are then successfully realized in a non‐Hermitian metasurface. Final, the efficient THz modulation is experimentally demonstrated, which exhibits modulation depth exceeding 70% and Off‐On‐Off switching cycle less than 9 ps in one LP while remains unaffected in another one. Compared with conventional THz modulation devices, the metadevice shows several figures of merits, such as a single frequency operation, high modulation depth, and ultrafast switching speed. The proposed theory and loss‐induced non‐Hermitian device are general and can be extended to numerous photonic systems varying from microwave, THz, infrared, to visible light.

## Graphical abstract

As a novel approach for efficient terahertz (THz) switching, the general theoretical framework and experimental verification of a non‐Hermitian metasurface hosting a pair of anti‐chiral exceptional points are proposed. Compared with conventional THz modulation devices, the metadevice shows several figures of merits, such as a single frequency operation, high modulation depth, and ultrafast switching speed.

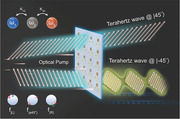

## 1 Introduction

Terahertz (THz) waves refer to electromagnetic radiation with oscillation frequencies in the range of 0.1–10 THz. This unique placement grants THz waves the advantage of combining microwave and optical characteristics, resulting in promising applications in high‐speed communication,^[^ ^1^, ^2^, ^3^ ^]^ imaging,^[^ ^4^, ^5^ ^]^ medical detection^[^ ^6^, ^7^ ^]^ and spectroscopy.^[^ ^8^, ^9^, ^10^ ^]^ However, the available natural materials for utilization in the THz range are quite limited. In that case, metamaterials composed of subwavelength meta‐atoms have gradually become one of the primary implementation solutions in THz modulation devices due to their compact structures, easy fabrication, and diverse functionalities, such as polarization conversion,^[^ ^11^, ^12^ ^]^ frequency filtering,^[^ ^13^, ^14^ ^]^ phase manipulation,^[^ ^15^, ^16^, ^17^ ^]^ holographic imaging,^[^ ^18^, ^19^, ^20^ ^]^ beam focusing,^[^ ^21^, ^22^, ^23^ ^]^ beam steering,^[^ ^24^, ^25^, ^26^ ^]^ and perfect absorption.^[^ ^27^, ^28^ ^]^ With the growing demand for compact, all‐optical, efficient and high‐speed systems in the next generation of wireless communication technology, THz switchable metasurfaces have gained extensive attention, which can be actively controlled by mechanical,^[^ ^29^, ^30^ ^]^ thermal,^[^ ^31^, ^32^, ^33^ ^]^ optical,^[^ ^34^, ^35^, ^36^ ^]^ electrical,^[^ ^37^, ^38^ ^]^ and magnetical^[^ ^39^ ^]^ methods. However, conventional THz switchable metadevices for efficient modulation concentrate on the Fano lineshape tailoring based on the temporal coupled‐mode theory (TCMT), resulting in a broadband resonance. Consequently, it is challenging to operate at the same frequency for both the dip and peak, making it difficult to achieve efficient modulation at a single frequency. This challenge may require the exploration of new physical mechanisms to provide resolutions.

Non‐Hermitian degeneracies, also known as exceptional points (EPs), existing in physical systems described by non‐Hermitian Hamiltonians that correspond to open physical systems considering the energy exchange between the system and the external environment, have become prominent in the realm of functional device, such as parity‐time (PT) symmetry‐broken lasers,^[^ ^40^, ^41^, ^42^ ^]^ ultra‐sensitive sensing,^[^ ^43^ ^]^ topological phase holography,^[^ ^44^, ^45^ ^]^ and unidirectional invisibility.^[^ ^46^ ^]^ Moreover, EPs can be constructed in non‐Hermitian metasurfaces by passively or actively scanning a given parameter space,^[^ ^47^, ^48^, ^49^, ^50^, ^51^ ^]^ which allows the investigation of the properties associated with PT‐symmetric, PT symmetry‐broken and EPs in non‐Hermitian metasurfaces.^[^ ^52^, ^53^, ^54^ ^]^ Recently, the topological properties of EPs have been thoroughly investigated in non‐Hermitian metasurfaces, including the degeneracy of eigenvalues and eigenstates,^[^ ^52^, ^53^, ^55^ ^]^ and the exceptional topological (ET) phase retardation.^[^ ^45^, ^56^ ^]^ Interestingly, the ET phase encircling EPs suggests that EPs can manipulate the dispersion of neighboring frequencies. Furthermore, research into chiral eigenstates have revealed the natural connection between the chirality of EPs and ±1 spin eigenstates.^[^ ^44^, ^57^ ^]^ The corresponding chiral eigenstates of EPs exhibit remarkable chiral dispersion characteristics.^[^ ^45^, ^53^ ^]^ However, as an important physical quantity in non‐Hermitian systems, the exploration of the combination of different chiral eigenstates remains insufficient. It is urgent to found a link between the chiral EPs and functional devices for efficient modulation.

In this work, we propose a theoretical framework to achieve efficient THz modulation at a single operating frequency. Based on the Pancharatnam‐Berry (PB) phase unitary transformation. the matrix of chiral transmission coefficients at the single operating frequency where *t*_rl_ intersects *t*_lr_ is transformed to ±45° linear polarization (LP) basis. Consequently, we establish a direct connection between the coupling variation of ±1 spin eigenstates and efficient modulation, which is enabled by the EPs of contrary chirality. As an implement of the theory, we construct a germanium (Ge)‐hybrid non‐Hermitian metasurface hosting a pair of anti‐chiral EPs. This non‐Hermitian system undergoes a loss‐induced phase transition from PT‐symmetry to broken PT‐symmetry, which corresponds to a process from coupling to decoupling of ±1 spin eigenstates. Initially, in the presence of PT‐symmetry, the single operating frequency exhibits the coupling of ±1 spin eigenstates accompanied with the asymmetric transmission of two orthogonal LP eigenstates |±45°〉. With the increased loss tuned by Ge, the system progressively reaches the broken PT‐symmetry region and the pair of anti‐chiral EPs gradually manifest and eventually merge. Since the EPs of contrary chirality merge at frequencies closing to the single operating frequency, the coupling channel between the ±1 spin eigenstates are closed. Then the ±1 spin eigenstates are decoupling and the asymmetric transmission of |±45°〉 LP is eradicated. Enabled by the coupling variation, we obtain asymmetric modulation in |±45°〉 LP. The co‐polarized transmission of |−45°〉 operates at a single frequency with a modulation depth over 70% and Off‐On‐Off switching cycle of less than 9 ps, while the co‐polarized transmission of |45°〉 LP remains unaffected. This work not only designs a efficient THz switching metadevice overcoming the challenge in Fano lineshape tailoring, but also develops the new physics of non‐Hermitian metasurfaces. We believe that this work provides a novel and universal principle for functional and switchable metadevices.

## 2 Results

### 2.1 Efficient LP Modulation via Coupling Variation of ±1 Spin States

With no loss of generality, we consider the matrix of chiral transmission coefficients at the intersection of *t*_rl_ and *t*_lr_, showing the same amplitude of *t*_lr_ and *t*_rl_ but differing in their phase. Here, we show the case of maximal coupling dispersion of *π* and can yet be perfectly eliminated by introducing the PB phase. This elimination is enabled by the conjugate phase retardation introduced by PB phase for *t*_rl_ and *t*_lr_.^[^ ^58^ ^]^ Since the cross‐polarization conversion efficiency in single‐layer metasurface typically does not exceed 1/2. The chiral transmission coefficients matrix can be defined as:

(1) $$T_{circ}=\left[\begin{array}{cc} \frac{1}{2}+\delta & \left(\frac{1}{2}-\delta \right)\times e^{-i\frac{\pi }{2}}\\ \left(\frac{1}{2}-\delta \right)\times e^{i\left(\frac{\pi }{2}-\alpha \right)} & \frac{1}{2}+\delta \end{array}\right]\;$$

where *δ* and *α* denoting the coupling variation and coupling dispersion between ±1 spin states, respectively. And spin states of +1(−1) represent circular polarization states with pure left (right)‐handed chirality, respectively. In the condition of *δ* = 0, ±1 spin states are coupling, whereas they are completely decoupling when *δ* = 1/2. In order to modulate the coupling dispersion, the PB phase unitary transformation can be employed. According to the initial coupling dispersion *π*, the unitary transformation from circular basis of ±1 spin states to linear basis of |±45°〉 LP is carried out. The Jones matrix for unitary transformation can be written as

(2) $$U\;=\frac{1}{\sqrt{2}}\;\left[\begin{array}{cc} 1 & -i\\ 1 & i \end{array}\right]$$

(3) $$R\;=\left[\begin{array}{cc} cos\left(\theta \right) & -sin\left(\theta \right)\\ sin\left(\theta \right) & cos\left(\theta \right) \end{array}\right]\;$$

where *U* and *R* are used for basis transformation and coordinate rotation, respectively. Noting that *θ* is set to be 45° according to the initial coupling dispersion *π*. Thus, the transmission matrix coefficients in the |±45°〉 linear basis can be obtained by the unitary transformation:

(4) $$\begin{array}{ccc} T_{|\pm 45^{\circ }\rangle } & = & R^{-1}\;U^{-1}T_{circ}UR\;\\ & & =\left[\begin{array}{cc} \frac{\delta }{2}+\frac{e^{-i\alpha }}{4}-\frac{\delta \times e^{-i\alpha }}{2}+\frac{3}{4} & \frac{i\left(e^{-i\alpha }-1\right)\left(2\delta -1\right)}{4}\\ \frac{i\left(e^{-i\alpha }-1\right)\left(2\delta -1\right)}{4} & \frac{3\delta }{2}-\frac{e^{-i\alpha }}{4}+\frac{\delta \times e^{-i\alpha }}{2}+\frac{1}{4} \end{array}\right] \end{array}$$

when coupling dispersion coefficient *α* = 0, the phase difference between *t*_lr_ and *t*_rl_ equals to *π*, indicating the maximal coupling dispersion condition. Then we obtain the target PB phase evolution trajectory, and the transformed transmission matrix in Equation 4 can be simplified as:

(5) $$T_{|\pm 45^{\circ }\rangle }^{trajectory}=\left[\begin{array}{cc} 1 & 0\\ 0 & 2\delta \end{array}\right]\;$$

**Figure 1:**
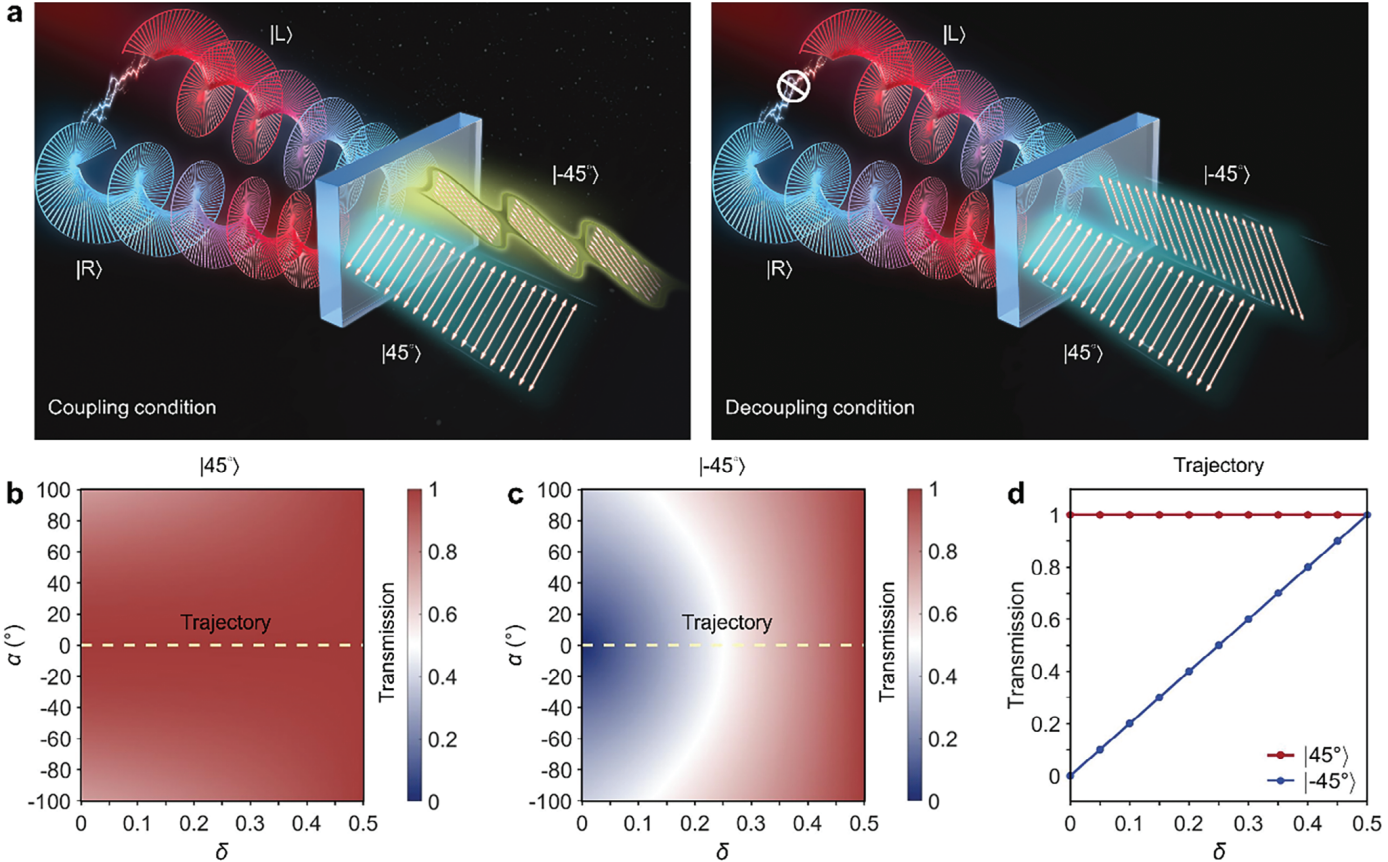
Design Principle of the efficient LP modulation enabled by coupling variation of ±1 spin states. a) Illustration of the PB phase device for efficient modulation based on the coupling variation of ±1 spin states and unitary transformation from circular basis of ±1 spin states to linear basis of |±45°〉 LP. b,c) Theoretically‐calculated phase diagram of co‐polarized transmission of b) |45°〉 and c) |−45°〉 LP in parameter space covered by *δ* ∈ [0, 0.5] and *α* ∈ [−100°, 100°]. The PB phase evolution trajectory for efficient modulation is obtained at *α* = 0, as indicated in b) and c) by shallow yellow dashed lines. d) The co‐polarized transmission of |±45°〉 LP extracted from the trajectory in b) and c).

Interestingly, the transmission matrix shows asymmetric modulation in |±45°〉 LP, as shown in **Figure** **1**a. In this case, the co‐polarized transmission of |−45°〉 LP can be modulated by adjusting the coupling variation coefficient *δ*, while there is no effect in |45°〉 LP. In order to observe the phase diagram of the co‐polarized transmission of |±45°〉 LP, a parameter space is defined by varying *δ* and *α* within the range of [0, 0.5] and [−100°, 100°] respectively. The corresponding phase diagram can be theoretically calculated using Equation 4, as depicted in Figure 1b,c. Interestingly, according to the Equation 5 in conjunction with the phase diagram, it is clear that the trajectory corresponding to *α* = 0, where the coupling dispersion is eliminated, is the most efficient for asymmetric modulation in |±45°〉 LP. The extracted co‐polarized transmission of |±45°〉 LP are illustrated in Figure 1d. Significantly, by implementing the PB phase and unitary transformation of the chiral transmission coefficients matrix located at the intersection of *t*_rl_ and *t*_lr_, the co‐polarized transmission of |−45°〉 LP is completely modulated, while the co‐polarized transmission of |45°〉 LP remains unaffected. Generally, in our phase diagram, the coupling condition of *δ* = 0 and *δ* = 1/2 correspond to physically anisotropic and isotropic systems.

### 2.2 Theoretical Proposal of ±1 Spin Eigenstates Coupling Variation Enabled by Loss‐Induced Anti‐chiral EPs

**Figure 2:**
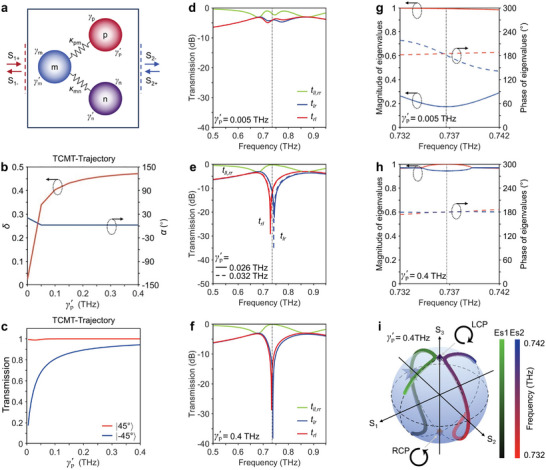
Theoretical proposal of ±1 spin eigenstates coupling variation enabled by loss‐induced anti‐chiral EPs. a) Schematic of the non‐Hermitian system supporting one *y*‐polarized modes and two *x*‐polarized modes that are coupled to two ports. TCMT‐calculated PB phase evolution trajectory evolves with non‐radiative loss rate *γ*′*_p_*, as indicated in b) varied *δ* and *α* and c) asymmetric transmission of |±45°〉 LP. The chiral transmission coefficients calculated from the theoretical TCMT model at *γ*′*_p_* = d) 0.005, e) 0.026 and 0.032, and f) 0.4 THz, indicating the coupling variation of ±1 spin eigenstates in the system. Both *t*_lr_ and *t*_rl_ reach almost 0 under completely decoupling condition in (f). The single operating frequency at 0.737 THz where *t*_lr_ intersects *t*_rl_ is highlighted in d–f) by black dashed lines. TCMT‐calculated eigenvalue spectrum of eigentransmission matrix at *γ*′*_p_* = g) 0.005, and h) 0.4 THz. Red lines correspond to one eigenvalue while blue lines correspond to another one. The single operating frequency highlighted in d–f) is highlighted by black dashed lines in (g–h). i) The TCMT‐calculated eigenstates versus frequency parametrically plotted on the Poincaré sphere at *γ*′*_p_* = 0.4 THz. The north pole of Poincaré sphere corresponds to +1 spin eigenstate, while the south pole of Poincaré sphere corresponds to −1 spin eigenstate.

It is worth noting that recent research into non‐Hermitian metasurfaces have revealed the natural connection between the chiral eigenstates and chiral EPs.^[^ ^44^, ^57^ ^]^ As a result, we anticipate that this discovery can be utilized to realize the coupling variation of ±1 spin eigenstates in the theoretical framework. Now we come to realize the PB phase evolution trajectory by a pair of loss‐induced anti‐chiral EPs, achieved by the coordination of three coupling resonators featuring orthogonal eigenstates as outlined in the TCMT and depicted in **Figure** **2**a. Here, “p” represents a resonator with *y*‐polarized eigenstate that couples with the incoming and outgoing waves, whereas “m” and “n” possess *x*‐polarized eigenstates and exclusively emit the *x*‐polarized wave. Defining the Hamiltonian of the three modes existing in the three coupling resonators as $H|\Psi_{i}\rangle =\hbar \omega_{i}\left|\Psi_{i}\right\rangle \left(i\;=\;p,m,n\right)$, respectively. Then the wave function of the system is $\left|\Psi \right\rangle =\Sigma c_{i}\left|\Psi_{i}\right\rangle$. To obtain the eigentransmission matrix of the three‐mode two‐port system, a parameter‐dependent non‐Hermitian Jones matrix of the designed non‐Hermitian system can be derived as

(6) $$H\;=\left[\begin{array}{ccc} \omega_{p}+i\left(\gamma_{p}+\gamma_{p}^{\prime }\right) & \kappa_{pm} & 0\\ \kappa_{pm} & \omega_{m}+i\left(\gamma_{m}+\gamma_{m}^{\prime }\right) & \kappa_{mn}\\ 0 & \kappa_{mn} & \omega_{n}+i\left(\gamma_{n}+\gamma_{n}^{\prime }\right) \end{array}\right]\;$$

where *ω*_*i*_ (*i*  =  *p*, *m*, *n*) =  2*π* *f_i_* (*i*  =  *p*, *m*, *n*) are the resonance (angular) frequency, *κ*_*ij*_ (*i*,*j*  =  *p*, *m*, *n*) describe the coupling strength of each two resonators, *γ*_*i*_ (*i*  =  *p*, *m*, *n*) and *γ*′*_i_* (*i  =  p, m, n*) represent the radiative loss rate and non‐radiative loss rate, respectively. Considering the incoming and outcoming wave at two ports defined as 1 and 2 with orthogonal polarization in *x* and *y*, which can be written as $|s^{j}\rangle =\left[s_{1x}^{j},s_{1y}^{j},s_{2x}^{j},s_{2y}^{j}\right]T\;\left(j\;=\;+,-\right)$, then the TCMT model can be defined as:^[^ ^59^, ^60^ ^]^

(7) $$\begin{array}{ccc} \frac{1}{2\pi }\;\frac{\partial a}{\partial t} & = & \;-i\left[\left(\begin{array}{ccc} f_{p} & \kappa_{pm} & 0\\ \kappa_{pm} & f_{m} & \kappa_{mn}\\ 0 & \kappa_{mn} & f_{n} \end{array}\right)-i\left(\begin{array}{ccc} \gamma_{p}^{\prime } & 0 & 0\\ 0 & \gamma_{m}^{\prime } & 0\\ 0 & 0 & \gamma_{n}^{\prime } \end{array}\right)\right]a\\ & & -\left(\begin{array}{ccc} \gamma_{p} & 0 & 0\\ 0 & \gamma_{m} & X_{mn}\\ 0 & X_{mn} & \gamma_{n} \end{array}\right)a+\left(\begin{array}{cccc} d_{1p} & 0 & d_{2p} & 0\\ 0 & d_{1m} & 0 & d_{2m}\\ 0 & d_{1n} & 0 & d_{2n} \end{array}\right)\left|s^{+}\right\rangle \end{array}$$

(8)

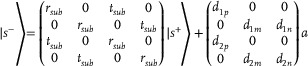

where *a*  = [*a_p_*, *a_m_*, *a_n_*]^*T*^  is a vector denoting the amplitude of the three modes in resonators. *X_ij_* (*i*,*j*  =  *p*, *m*, *n*) represent the far‐field coupling between “*i*” mode and “*j*” mode, *d*_*ki*_ (*k*  =  1, 2; *i*  =  *p*, *m*, *n*) are the coupling coefficients between incoming light and radiative modes “p”, “m” and “n” at port *k*  =  1, 2, and *r*_sub_ and *t*_sub_ constitute the background scattering matrix between the ports with the disappearance of resonance in the far field.

Utilizing standard TCMT theory in condition of energy conservation and time‐reversal symmetry, the eigentransmission matrix can be obtained from the joint solution of Equations 7 and 8.

(9) $$T_{lin}=\left[\begin{array}{cc} t_{xx} & t_{xy}\\ t_{yx} & t_{yy} \end{array}\right]\;=\left[\begin{array}{cc} s_{2x}^{-}/s_{1x}^{+} & s_{2x}^{-}/s_{1y}^{+}\\ s_{2y}^{-}/s_{1x}^{+} & s_{2y}^{-}/s_{1y}^{+} \end{array}\right]\;$$

then the four chiral transmission coefficients can be obtained from the eigentransmission matrix by basis transformation using matrix *U*.

(10) $$\begin{array}{ccc} \left[\begin{array}{cc} t_{ll} & t_{lr}\\ t_{rl} & t_{rr} \end{array}\right] & = & \;U\times T_{lin}\times U^{-1}\;\\ & & =\frac{1}{2}\;\left[\begin{array}{cc} t_{xx}+t_{yy}+i\left(t_{xy}-t_{yx}\right) & t_{xx}-t_{yy}-i\left(t_{xy}+t_{yx}\right)\\ t_{xx}-t_{yy}+i\left(t_{xy}+t_{yx}\right) & t_{xx}+t_{yy}-i\left(t_{xy}-t_{yx}\right) \end{array}\right] \end{array}$$

where *t*_ij_ are defined as the complex transmission coefficients for *i*‐polarized light outgoing with *j*‐polarized light incoming, *i*, *j* ∈ {*x*, *y*, *l*, *r*}.

Since the theoretical derivation of the chiral transmission coefficients matrix from the non‐Hermitian system, precise adjustment of the theoretical parameters in Equations 7 and 8 is essential to achieve the PB phase evolution trajectory. To extract and observe the trajectory in the TCMT model, the Equation 9 is first used to obtain the eigentransmission matrix. Then the chiral transmission matrix can be obtained by Equation 10. Subsequently, the single operating frequency corresponding to the intersection of *t*_lr_ and *t*_rl_ can be found. Finally, the coupling variation *δ* and coupling dispersion *α* can be extracted at the single frequency through the Equation 1, and the transmission of |±45°〉 LP can be extracted through the Equation 4. As depicted in Figure 2b,c, *α* = 0 signifies the elimination of the coupling dispersion, while the coupling variation *δ* has led to the asymmetric modulation in |±45°〉 LP. This indicates that the PB phase evolution trajectory for efficient and asymmetric modulation shown in Figure 1 has been established in the non‐Hermitian system. Notably, the trajectory can be completely evolved by tuning just one parameter, specifically the non‐radiative loss rate *γ*′*_p_*, thus facilitating convenient implementation of ultrafast switching within the system.

Accordingly, the coupling variation of ±1 spin eigenstates in the system are depicted in Figure 2d–f. The chiral transmission coefficients have been calculated from the theoretical TCMT model at *γ*′*_p_* = 0.005, 0.026 and 0.032, and 0.4 THz. The single operating frequency at 0.737 THz where *t*_lr_ intersects *t*_rl_ is highlighted by black dashed lines. Initially, with the low value of *γ*′*_p_*, the chiral transmission coefficients *t*_rl_ and *t*_lr_ are far greater than 0 at their intersection, which corresponds to *δ* approaching 0 and indicates the strong coupling between ±1 spin eigenstates. Finally, with the high value of *γ*′*_p_*, the chiral transmission coefficients *t*_rl_ and *t*_lr_ are almost simultaneously reaching 0, which corresponds to *δ* = 1/2 and indicates the completely decoupling between ±1 spin eigenstates. Furthermore, we have extracted the eigenvalue spectrum of the eigentransmission matrix in Equation 9 at *γ*′*_p_* = 0.005 and 0.4 THz, as illustrated in Figure 2g,h. Red lines correspond to one eigenvalue while blue lines correspond to another one. The single operating frequency highlighted in Figure 2d–f is highlighted by black dashed lines in Figure 2g,h. The eigenvalues have displayed immense disparities in the initial coupling condition at *γ*′*_p_* = 0.005 THz, while eventually degenerate at frequencies closing to 0.737 THz in the completely decoupling condition at *γ*′*_p_* = 0.4 THz. In addition, the TCMT‐calculated eigenstates versus frequency are parametrically plotted on the Poincaré sphere, as depicted in Figure 2i. The colorbar on the left corresponds to one eigenstate namely “Es1” while the colorbar on the right corresponds to another one namely “Es2”. And the colorbar has clarified the range of frequency on the right side. The eigenstates at frequencies closing to the single operating frequency almost reach the north and south poles, indicating the nearly merging of two anti‐chiral EPs. Since the EPs of contrary chirality merge at frequencies closing to the single operating frequency, the coupling channel between the ±1 spin eigenstates are closed. Then the ±1 spin eigenstates are completely decoupling at the single operating frequency, which corresponds to *δ* = 1/2. Consequently, the asymmetric transmission of |±45°〉 LP is eradicated.

### 2.3 Implement to Photonic Systems for efficient THz Modulation

**Figure 3:**
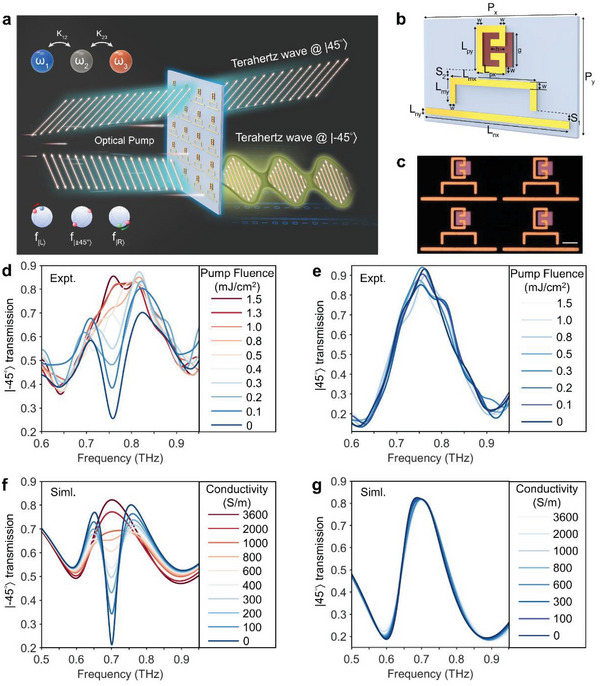
Experimental and simulated co‐polarized transmission spectra of |±45°〉 LP of a non‐Hermitian metasurface that supports the coupling variation of ±1 spin eigenstates. a) Illustration of the non‐Hermitian metasurface hosting a pair of anti‐chiral EPs for efficient modulation and ultrafast photoswitching at picosecond timescale. The metasurface is hybrid with the 300‐nm‐thick amorphous Ge layer to generate perturbations via photocarriers excited by the optical pump. b) The geometric parameters of unit cell are *P*_x_ = 160, *P*_y_ = 98, *L*_nx_ = 150, *L*_ny_ = 7, S_1_ = 4.5, *L*_mx_ = 75, *L*_my_ = 25, w = 6, S_2_ = 4, *L*_px_ = 28, *L*_py_ = 43, g = 30, h = 14, with unit of µm. c) Microscopic image of the Ge‐hybrid non‐Hermitian metasurface, the scale bar is 30 µm. Experimental demonstration of co‐polarized transmission spectra of d) |−45°〉 and e) |45°〉 LP versus pump fluence. Simulated co‐polarized transmission spectra of f) |−45°〉 and g) |45°〉 LP versus simulated Ge conductivities.

In order to implement the aforementioned theoretical framework for efficient THz switching, we construct the switchable Ge‐hybrid non‐Hermitian metasurface. The concept and design of the metasurface are depicted in **Figure** **3**a. It is composed of periodically arranged meta‐atoms consisting of three resonators that are orthogonally oriented. The split ring resonator (SRR) aligned along the *y*‐direction, functions as an inductance capacitance (LC) resonance corresponding to the “p” resonator with a *y*‐polarized eigenstate. The orthogonally oriented SRR and cut‐wire (CW) with *x*‐polarized eigenstates are distributed near and far from the *y*‐oriented SRR, which represent the “m” and “n” resonators and act as LC resonance and dipole moment, respectively. To actively control the non‐radiative loss of the “p” resonator, the 300‐nm‐thick amorphous Ge layer is deposited into the gaps of the *y*‐oriented SRR, serving as photon‐induced perturbations. The design principle of the non‐Hermitian system based on the TCMT is provided in the Supporting Information. The geometric parameters of the meta‐atoms are *P*_x_ = 160, *P*_y_ = 98, *L*_nx_ = 150, *L*_ny_ = 7, S_1_ = 4.5, *L*_mx_ = 75, *L*_my_ = 25, w = 6, S_2_ = 4, *L*_px_ = 28, *L*_py_ = 43, g = 30, h = 14, with unit of µm, as shown in Figure 3b. The fabricated metasurface is captured with reflective optical microscopy, as shown in Figure 3c.

In the following, we demonstrate the application of our metadevice in switching the co‐polarized transmission of |±45°〉 LP, which is efficient and single frequency operation. We start with an experimental verification of the theoretical proposal via perturbations of photo‐carriers excited by femtosecond optical pulses with various optical pump powers. Our homemade optical pump THz‐probe (OPTP) system is used to obtain THz transmission spectra through the metasurface at different pump power, which enables us to observe the changes of the co‐polarized transmission spectrum in |±45°〉 LP as a function of pump power in Figure 3d,e. Notably, the |−45°〉 LP undergoes spectral modulation with an amplitude ranging from 0.26 to 0.86 at 0.76 THz, while the |45°〉 LP remains nearly unaffected.

To validate the observed transmission spectrum in our experiment, we conduct finite element method (FEM) simulations using CST Studio Suite. These simulations accurately compute the results based on the device and material parameters utilized in our experiments. The results presented in Figure 3f,g exhibit a remarkable agreement between the simulated transmission amplitude and the experimental data. Of particular interest is the single frequency operation in |−45°〉 LP, while the co‐polarized transmission of |45°〉 LP remains nearly unaffected. However, a slight deviation of ≈0.06 THz is observed in the resonance peak of the simulation compared to the experimental findings. This deviation could be attributed to several factors. Primarily, it may arise from disparities in the refractive index of the *z*‐cut quartz substrate. Additionally, minor differences in meta‐atom sizes between the optimized simulated ones and the fabricated ones could lead to random shifts in the resonance frequency of each meta‐atom. Furthermore, the actual Ohmic loss in the gold could potentially be higher than the ideal value used in our simulations (4.56 × 10^7^ S m^−1^). This higher Ohmic loss resulting from the fabrication process could weaken the strength of the resonance, causing the shift in resonance frequency. Lastly, the *z*‐cut quartz substrate may not be adequately cleaned after the fabrication process, especially when the spacer layer (PMMA) is spin‐coated around the metasurface. The residual PMMA may introduce additional loss for the resonant current in the metasurface. While we acknowledge the importance of addressing this deviation, it is crucial to note that it does not significantly impact the modulation effect and the observation of the singular phenomena. The shift in resonance frequency simply translates to a corresponding shift in the parameter space.

**Figure 4:**
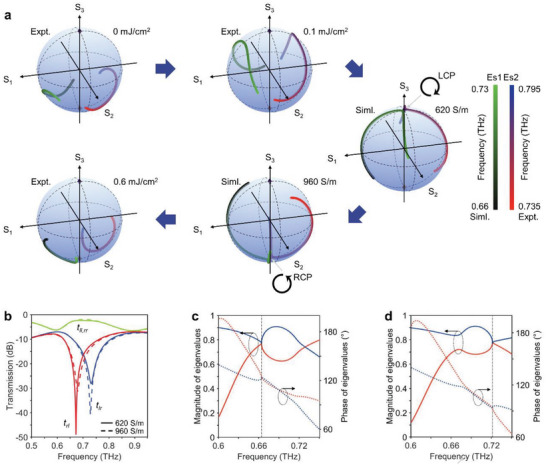
Optical access to eigenstates at the pair of anti‐chiral EPs. a) Experimentally‐extracted and numerically‐simulated eigenstates with increasing perturbation of photoconductivity introduced by the 300‐nm‐thick Ge layer are parametrically plotted on the Poincaré sphere versus frequency. b) Spectral dependence of the chiral transmission coefficients matrix. A pair of anti‐chiral EPs is observed at simulated Ge conductivities of 620 and 960 S m^−1^, corresponding to left‐handed and right‐handed chirality, respectively. Simulated magnitude and phase of eigenvalues of the eigentransmission matrix. The degeneracy occurs at c) the frequency of 0.664 THz corresponding to the +1 spin eigenstate |L〉 and d) the frequency of 0.722 THz corresponding to the −1 spin eigenstate |R〉, as indicated by black dashed lines.

Afterward, in order to observe the pair of anti‐chiral EPs in the non‐Hermitian metasurface, optical access has been carried out both experimentally and numerically. This encompasses an analysis of the eigenvalues and eigenstates of the non‐Hermitian three‐mode two‐port system under various optical pump powers in the experiment and swept Ge conductivities in the simulation. To illustrate the eigenstates coalescing behavior, numerically‐calculated and experimentally‐extracted polarization eigenstates are parametrically plotted on the Poincaré sphere. For the purpose of clarity, we showcase the variation of polarization eigenstates versus frequency corresponding to increasing non‐radiative loss of “p” resonance, denoted by blue arrows, associated with different values of optical pump powers (or Ge conductivities). As depicted in **Figure** **4**a, the colorbar on the left corresponds to one eigenstate namely “Es1” while the colorbar on the right corresponds to another one namely “Es2”. Moreover, the colorbar has clarified the range of frequency in both simulation and experiment, on the left and right side, respectively. Except for the pair of anti‐chiral EPs, the polarization eigenstates exist in pairs and symmetrically approach the poles. As the anti‐chiral EPs are successively approached in parameter space, the paired polarization eigenstates gradually move toward the north pole on the Poincaré sphere, subsequently coalesce into the +1 spin eigenstate |L〉, followed by the −1 spin eigenstate |R〉. Due to its sensitivity, the presence of the anti‐chiral EPs in the experiment is challenging to rigorously verify. Nevertheless, we conclude that EPs corresponding to the left‐handed and right‐handed chirality sequentially emerge at Ge conductivities of 620 and 960 S m^−1^ in our simulation. Figure 4b illustrates the spectral singularity point of *t*_rl_ and *t*_lr_ corresponding to the left‐handed and right‐handed chirality, demonstrating the existence of the pair of anti‐chiral EPs. The singular topological characteristic of the pair of anti‐chiral EPs is further affirmed by the degeneracy of the numerically calculated magnitude and phase of eigenvalues of eigentransmission matrix at frequencies of 0.664 THz and 0.722 THz, as indicated by blue dashed lines in Figure 4c,d, respectively.

### 2.4 Experimental Characterization of Ultrafast THz Wave Switching

**Figure 5:**
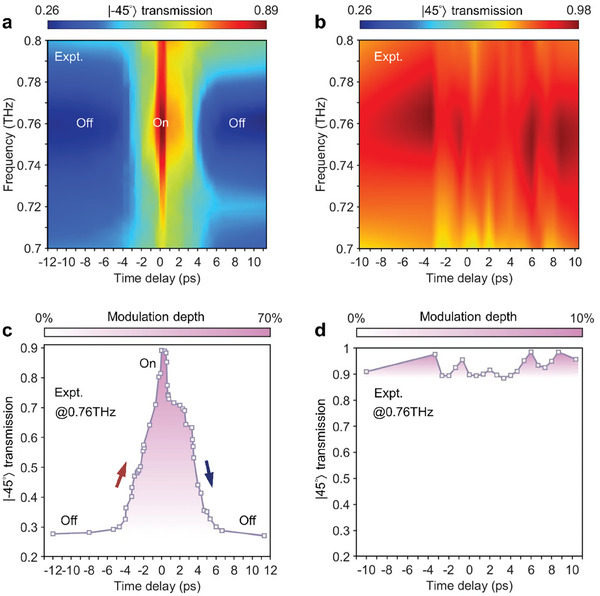
Measured transient dynamic of the non‐Hermitian metasurface for efficient THz switching. Colormap showing the co‐polarized transmission of a) |−45°〉 and b) |45°〉 LP against frequency and pump‐probe time delay over the entire ultrafast Off–On–Off photo‐switching cycle. The co‐polarized transmission and normalized modulation depth of c) |−45°〉 and d) |45°〉 LP extracted at the frequency of 0.76 THz from the experimental results in (a) and (b), respectively.

In the following, we demonstrate the practical implementation of our metadevice in switching the co‐polarized transmission of the |±45°〉 LP on a picosecond timescale, a crucial aspect in THz spectroscopy and ultrafast photonics. The strong interaction of transient dynamic materials with resonances is of vital importance in realizing ultrafast control of EP photonic metadevices. The presence of defects in semiconductors provides an additional channel to trap free carriers with faster recombination rate, while the concomitant appearance of localized energy states leads to the poor photoconductivity.^[^ ^61^, ^62^, ^63^ ^]^ Such an ultrafast free carrier relaxation is treated as a transient perturbation on the non‐Hermitian metasurface. The unique feature of swift relaxation in the 300‐nm‐thick Ge film occurs with sub‐picosecond lifetime, as shown in Figure S1 (Supporting Information). Pumping the THz metadevice with an optical beam of wavelength 800 nm (1.55 eV), the transient dynamics of efficient THz switching can be unveiled by the OPTP technique. We commence with an experimental characterization of the co‐polarized transmission of |−45°〉 LP through perturbations of photo‐carriers induced by femtosecond optical pulses. The time‐resolved transmission spectra dependent on frequency, as a function of time delay, is depicted in **Figure** **5**a. Given the time‐dependent switching behavior, we select a pump‐probe time delay of 0 picosecond, corresponding to the peak response. Notably, the metadevice provides a high‐contrast binary‐level switching (Off–On), which enables an Off–On–Off switching cycle in less than 9 ps. Defining the modulation depth as *D*  = (*t*_pump_ − *t*_nopump_) /*t*_pump_ × 100%, it becomes apparent that the modulation of the co‐polarized transmission of |−45°〉 LP occurs, ranging from 0.26 to 0.89 in amplitude with a maximum modulation depth of 70% at 0.76 THz. In comparison, the co‐polarized transmission of |45°〉 LP is also measured. Evidently, the amplitude of |45°〉 LP exhibits a modulation depth of less than 10%, indicating nearly unaffected.

To further quantify the efficient switching functionality, Figure 5c precisely monitors the time dependence of the ultrafast switching process, depicted by the co‐polarized transmission of |−45°〉 LP at 0.76 THz. The co‐polarized transmission remains unaltered prior to −4 ps, registering at 0.26; subsequently, a discernible increase to 0.89 is clearly observed at 0 ps. This then recuperates to the initial value at 4.5 ps and sustains a constant level with no significant variation thereafter. Additionally, a 70% modulation depth within the 9 ps switching cycle at 0.76 THz in |−45°〉 LP is also quantitatively determined in Figure 5c. Correspondingly, the same assessment is applied to the |45°〉 LP as depicted in Figure 5d. The co‐polarized transmission of |45°〉 LP remains nearly unaffected, while co‐polarized transmission of |−45°〉 LP is considerably affected by the presence of free carriers, indicating a remarkably ultrafast, efficient and single frequency switching operation mechanism.

## 3 Conclusion

In this work, we have proposed a novel theoretical framework with its metadevice verification founding a link between the chiral EPs in non‐Hermitian physics and functional devices for efficient modulation. Demonstrating this theory, the non‐Hermitian metasurface exhibits exceptional performance with a single frequency operation, high modulation depth, and ultrafast switching speed. We start with the establishment of a general PB phase evolution phase diagram, which reveals a direct connection between the coupling variation of ±1 spin states and efficient modulation. Through the unitary transformation of the chiral response, results show asymmetric modulation in |±45°〉 LP, in which the co‐polarized transmission of |−45°〉 LP undergoes complete modulation while the co‐polarized transmission of |45°〉 LP remains unaffected. To realize this general phase diagram, we construct a three‐mode two‐port non‐Hermitian system described by the TCMT, containing one *y*‐polarized eigenmode and two *x*‐polarized eigenmodes. After testing the PB phase evolution trajectory enabled by a pair of loss‐induced anti‐chiral EPs, we apply the theoretical framework to a non‐Hermitian metasurface that meticulously designed and fabricated based on physical parameters in the theoretical three‐mode two‐port system. To achieve the loss‐induced PT phase transition, the non‐Hermitian metasurface is hybrid with the Ge layer functioned as an active switching knob. Through a transient loss with sub‐picosecond lifetime introduced by femtosecond optical pulses, the efficient modulation in co‐polarized transmission of |−45°〉 LP is successfully characterized, featuring its merits of a single frequency operation at 0.76 THz, modulation depth over 70%, and Off–On–Off switching cycle less than 9 ps, while the co‐polarized transmission of |45°〉 LP remains unaffected.

Our work not only designs a non‐Hermitian THz metasurface that overcomes the challenge in Fano lineshape tailoring, but more importantly, our proposed theoretical framework is generic and develops the new physics of non‐Hermitian metasurfaces, which can be easily applied to other frequency regions due to the easy access to electromagnetic loss. It can also serve as a crucial tool for investigating transient dynamic phenomena related to non‐Hermitian chiral degeneracies and designing a variety of metadevices with innovative functionalities. Our work facilitates practical applications of PT‐symmetric photonics devices and paves the way for extensive research prospects within the interdisciplinary field of nonlinear optics and topological photonics.

## 4 Experimental Section

### Numerical Simulations

The FEM in CST Studio Suite conducts numerical simulations. The simulation entails configuring the gold material as a lossy metal with a conductivity of 4.56 × 10^7^ S m^−1^, setting the quartz substrate as a normal dielectric with a permittivity of 3.9204. The Ge material, possessing a permittivity of 16, exhibits a time‐dependent conductivity derived from experimental results, while the input excitation signal is normalized using the measured THz time‐domain pulse. Boundaries are imposed in the *x* and *y* directions of the unit cell, with open boundary conditions governing the *z* direction. A frequency‐domain solver is utilized to extract the transmission spectra.

### Metadevice Fabrication

The fabrication process involved constructing a Ge‐hybrid non‐Hermitian metasurface on a commercially available *z*‐cut quartz substrate with a 2 mm thickness using traditional microfabrication methods. An initial 300 nm gold layer was e‐beam evaporated, preceded by a 10 nm chromium adhesion layer. Subsequently, the structured design was created through standard photolithography techniques with a lithographic mask, followed by a lift‐off procedure. Next, a 300‐nm amorphous Ge layer was sputtered onto the substrate utilizing magnetron sputtering post the second photolithography and lift‐off process. Ultimately, the organized Ge‐hybrid metal metasurface structure, measuring 6 mm × 6 mm, was fabricated.

### Experimental Measurement

The characterization of the THz transmission of the Ge‐hybrid non‐Hermitian metadevice was conducted using a homemade OPTP system. A Ti sapphire regenerative amplifier system generated a femtosecond pulse with a 1 kHz repetition rate at an 800 nm central wavelength to induce THz generation and detection from the nonlinear effect of a 1‐mm‐thick ZnTe 〈110〉 crystal. Four linearly polarized transmission signals were measured by rotating the sample and linear polarizer. The spectral information of the complex THz transmission was extracted through Fourier transform of the time‐domain signal. Subsequently, the substrate data was normalized, which can be expressed as *T* (ω) = *E_s_* (ω)/*E_R_* (ω). The measurement of the ultrafast THz modulation was accomplished by adjusting the relative position between the THz signal and the optical pump pulse.

## Conflict of Interest

The authors declare no conflict of interest.

## Author Contributions

Z.Y. and Y.H. conceived the idea. Z.Y., Y.H., and W.H. designed the experiment and performed active measurements and all the simulations. Z.Y., S.W., and Z.R. fabricated the samples. Z.Y., W.H., and S.H. discussed and analyzed the measured data. X.C., Y.H., and T.J. supervised the theory and the measurements. Z.Y. prepared the manuscript with inputs from T.J. and Y.H.

## Supporting information

Supporting Information

## Data Availability

The data that support the findings of this study are available from the corresponding author upon reasonable request.
