# Supplementary material for: Creating Anti‐Chiral Exceptional Points in Non‐Hermitian Metasurfaces for Efficient Terahertz Switching
Source: Adv Sci (Weinh). 2024 May 17;11(28):2402615. doi: 10.1002/advs.202402615 (PMC11267315; doi:10.1002/advs.202402615)
Supplement: Supplementary file 1 — Supporting Information [file ADVS-11-2402615-s001.docx]

***Supporting Information***

**Creating anti-chiral exceptional points in non-Hermitian metasurfaces for efficient terahertz switching**

*Zhongyi Yu^1^, Weibao He^1^, Siyang Hu^1^, Ziheng Ren^1^, Shun Wan^1^,* *Xiang’ai Cheng^1^, Yuze Hu^2,*^, Tian Jiang^2,*^*

^1^College of Advanced Interdisciplinary Studies, National University of Defense Technology, Changsha 410073, P. R. China

^2^Institute for Quantum Science and Technology, College of Science, National University of Defense Technology, Changsha 410073, P. R. China

Correspondence: Professor Yuze Hu, E-mail: hyz_yj@sina.com

Correspondence: Professor Tian Jiang, E-mail: tjiang@nudt.edu.cn

**1. OPTP measurements for an amorphous Ge film**


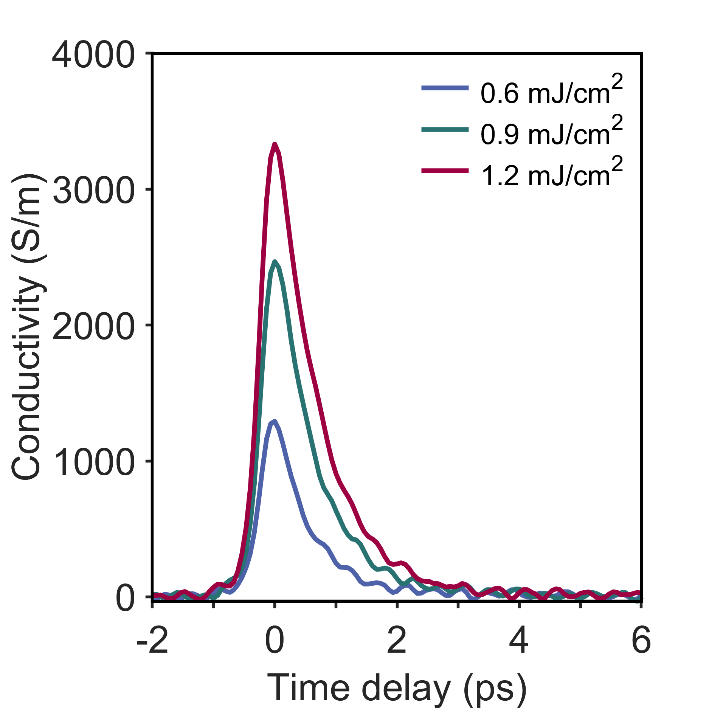


**Figure S1. Sub-picosecond time scale photocarrier dynamics of the amorphous Ge film.** The solid curves indicate the change in THz transmission amplitude measured under the influence of optical pumping at various fluences.

**2. Simulation results of the anti-chiral EPs in the designed non-Hermitian metasurface**


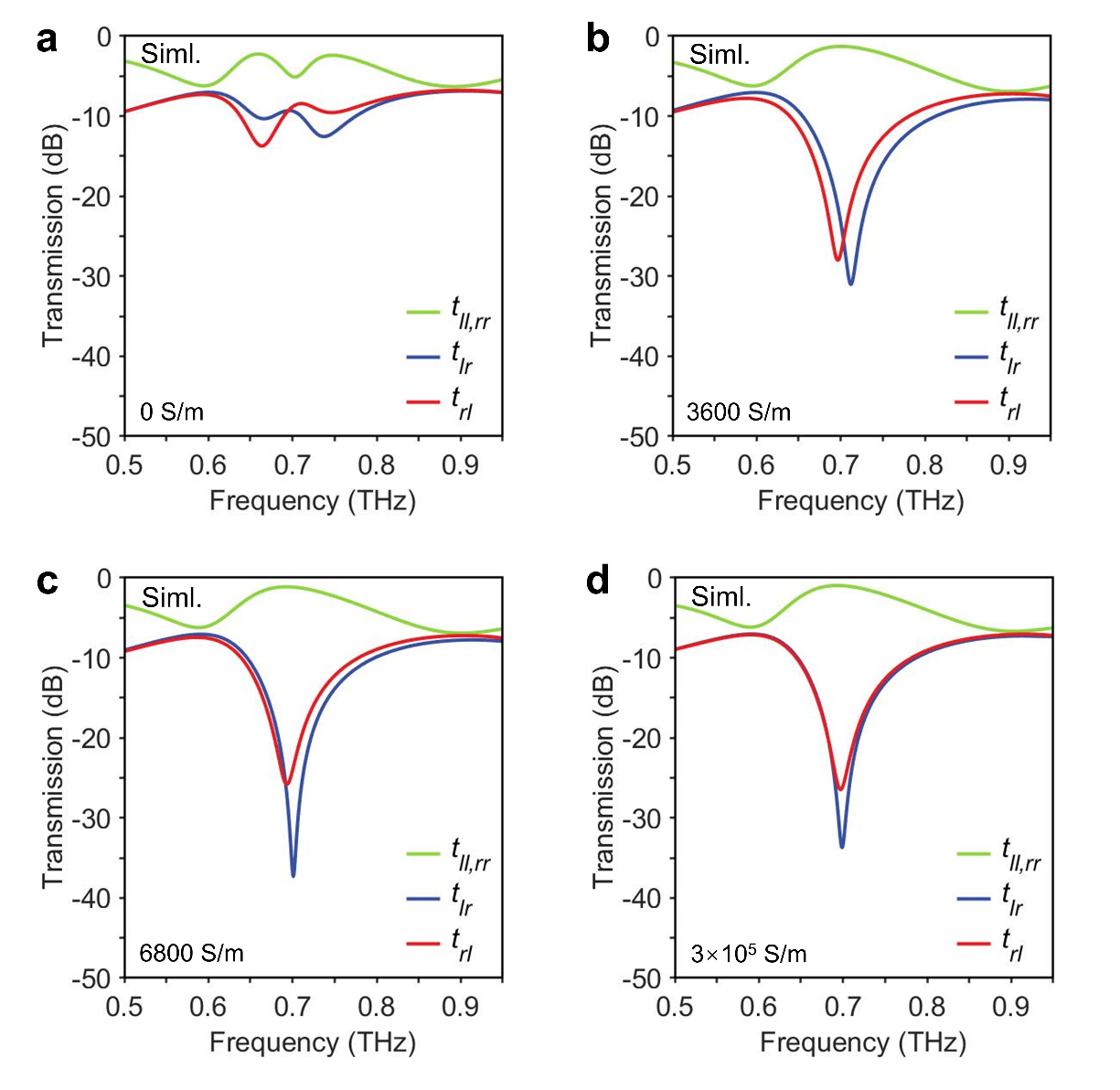


**Figure S2. Simulated chiral transmission coefficients in the designed non-Hermitian metasurface.** The cross-polarized chiral transmission coefficients at different conductivity of Ge of a) 0, b) 3600, c) 6800, and d) 3 × 10^5^ S/m.

The conductivity of Ge typically does not exceed 3600 S/m, which entails a slight constraint on the decoupling effect of anti-chiral EPs towards circular polarization states with left-handed and right-handed chirality, thus slightly constraining the modulation depth of the non-Hermitian metasurface. Nevertheless, in simulations, it may be advantageous to set it higher to observe the performance of the anti-chiral EPs in the non-Hermitian metasurface. Moreover, as the conductivity of Ge reaches 3600 S/m, the frequencies at which the minimum values of *t_rl_* and t*_lr_* occur are already quite close, and the amplitude at the intersection of *t_rl_* and t*_lr_* is relatively low as well.

**3. Design principle of the non-Hermitian system based on the TCMT.**

The non-Hermitian metasurface consists of three discrete resonators: upper split ring resonator (SRR), middle SRR, and lower cut-wire (CW), corresponding to the resonant modes “p”, “m”, and “n”, respectively. According to the orientation, the eigenmode of “p” is *y-*polarized, while the other two are *x-*polarized, which are consistent with the three modes in TCMT. Subsequently, the optimization of structural parameters has been carried out to align the coupling parameters within the metasurface. For instance, modifying S_1_ can tune the coupling strength between modes “p” and “m”, corresponding to *κ_pm_* in the TCMT. Likewise, modifying S_2_ can tune the coupling strength between modes “m” and “n”, corresponding to *κ_mn_* in the TCMT. Varying the aperture size of the upper and middle SRR can tune the radiative loss rate of mode “p” and of mode “m”, while modifying the length of the lower CW can tune the radiative loss rate of mode “n”. Additionally, modifying the conductivity of Ge embedded in the upper SRR can tune the non-radiative loss rate . The parameter adjusted in the TCMT theoretical model corresponds to the conductivity of Ge in simulations and the pump fluence of femtosecond optical pulses used for pumping Ge in experiments.
